# Orbital Electrowetting: From Continuous Droplet Transport to Programmable Microfluidics

**DOI:** 10.1002/adma.73401

**Published:** 2026-05-14

**Authors:** Jie Tan, Jiayu Du, Dong Lv, Yikui Gao, Dongyue Jiang, Zuankai Wang, Wenjie Liu, Chi Yan Tso

**Affiliations:** ^1^ School of Energy and Environment City University of Hong Kong Hong Kong China; ^2^ Key Laboratory of Ocean Energy Utilization and Energy Conservation of Ministry of Education Dalian University of Technology Dalian China; ^3^ Department of Mechanical Engineering The Hong Kong Polytechnic University Hong Kong China; ^4^ School of Energy and Environmental Engineering University of Science and Technology Beijing Beijing China

**Keywords:** droplet transport, electrowetting‐on‐dielectric, microfluidics, surface tension

## Abstract

Droplet transport on solid surfaces underpins applications in microfluidics, thermal management, and water harvesting. Conventional electrowetting (CEW) has enabled digital droplet control, but reliance on numerous individually addressed electrodes, complex driving circuitry, and step‐wise motion has limited scalability and practical deployment. Orbital electrowetting (OEW) has recently been introduced as a complementary paradigm, where asymmetric electrowetting forces and electrostatic energy gradients drive droplets into continuous, high‐speed motion along predefined orbital pathways using only a minimal number of electrodes and simple global excitation. In this perspective, the physical mechanisms of OEW are summarized, with emphasis on how surface wettability, electric field distribution, and orbital geometry govern droplet speed, stability, and confinement. Key challenges are identified, including the current lack of robust bidirectional and position‐resolved control in complex networks. Future directions and application opportunities for OEW‐enabled platforms are outlined, such as programmable microfluidics with low overhead wiring, defogging and self‐cleaning of photovoltaic modules, enhancement of condensation heat transfer, and atmospheric water harvesting when combined with passive radiative cooling surfaces. It is argued that, as these challenges are addressed, OEW will substantially expand the electrowetting toolbox for next‐generation droplet manipulation and water‐energy technologies.

## Graphical abstract

This work comprehensively summarizes the mechanisms, recent advances, potential applications, and key challenges of orbital electrowetting. It highlights that integrating orbital electrowetting with conventional electrowetting is required to enable complete digital‐microfluidic workflows while simplifying platform architecture. It further emphasizes that future efforts should achieve bidirectional droplet transport and integrate with other technologies to enable a broader range of applications.

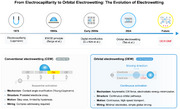

## 1 Introduction

**FIGURE 1:**
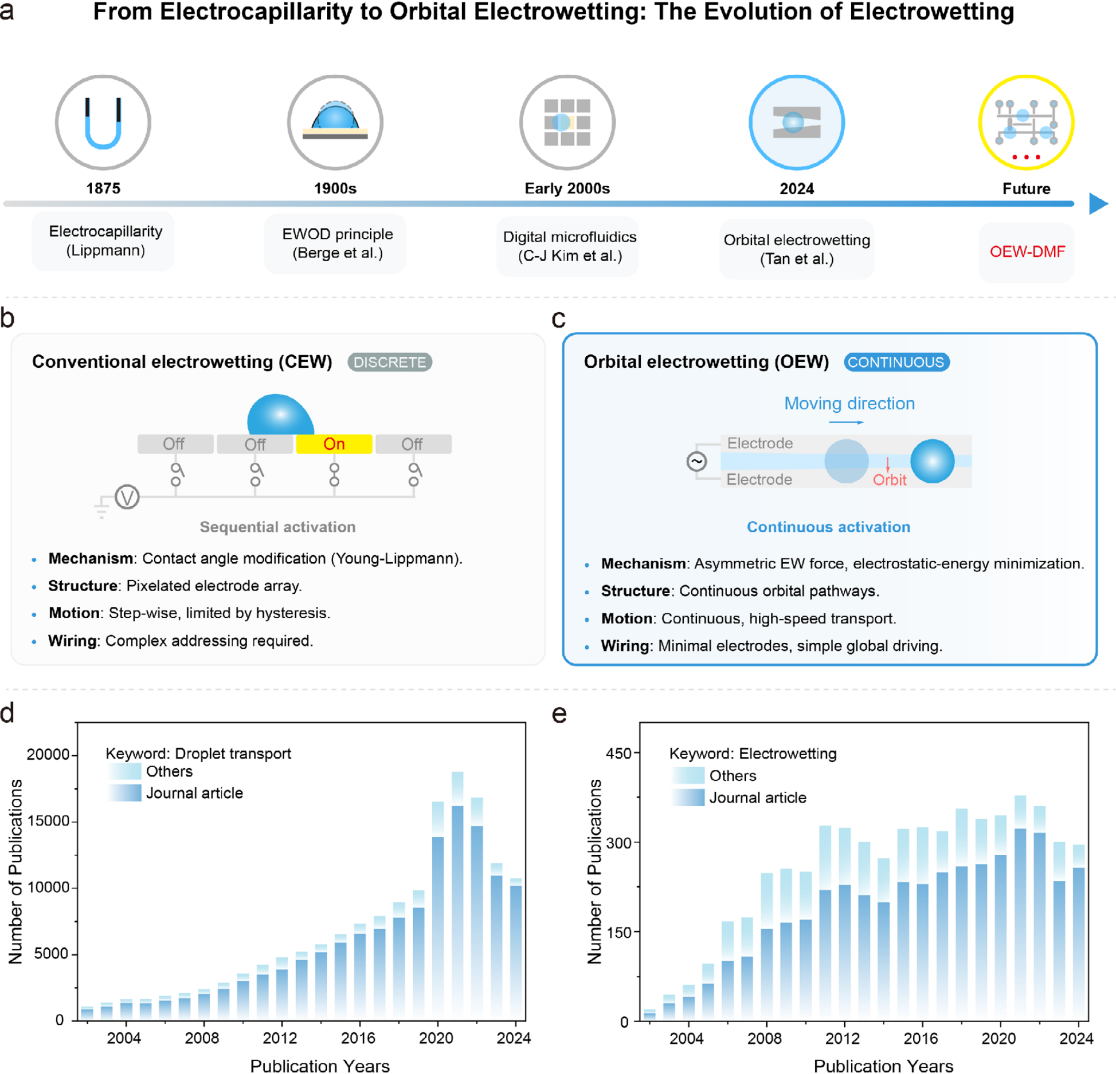
Evolutionary timeline of electrowetting in microfluidics from its inception in 1875 to the 21st century. (a), The evolution of electrowetting. (b), Principle and structural design of CEW droplet transport, illustrating the use of electrode arrays and complex driving circuitry. (c), Principle and structural design of OEW droplet transport, highlighting continuous droplet transport and simplified circuitry. Number of publications over the years related to droplet transport (d) and electrowetting (e), according to our set of keywords (*Source*: lens.org, 2002 to 2024).

The ability to precisely control surface droplets is of critical importance in a wide range of disciplines, including microfluidics [1, 2, 3], surface photothermal modulation [4], heat transfer [5, 6], energy harvesting [7, 8], and water collection [9, 10, 11, 12]. Existing strategies for achieving droplet manipulation rely on diverse actuation mechanisms such as surface topography [13], charge gradients [14], photopyroelectric effects [15], and magnetic field‐induced forces [16]. Among these, electrowetting has garnered significant attention due to its advantages, including structural simplicity [17], low energy consumption [18], fast response speed [19], and high programmability [20]. Electrowetting is a technique that modulates the solid–liquid interfacial tension by applying an electric potential, thereby enabling controlled deformation [21, 22] and motion of liquid droplets [23, 24]. Figure 1a summarizes the historical development of electrowetting and its evolution from fundamental observations to modern microfluidic applications. The phenomenon of electrowetting, specifically the behavior of mercury and other liquids on variably charged surfaces, was first systematically explained by Gabriel Lippmann as early as 1875 [25]. The direct contact between mercury droplets and electrodes often leads to electrolysis, resulting in the instability of the electrowetting phenomenon [26]. This issue was resolved with the introduction of a dielectric layer, which separates the aqueous solution droplet from the electrode [27]. The dielectric layer effectively prevents electrolysis and significantly expands the range of applied electric potential from a few volts to hundreds of volts [8]. This configuration, known as electrowetting‐on‐dielectric (EWOD) [28], distinguishes itself from conventional electrowetting on bare electrodes. In 1993, Bruno Berge integrated Young's wetting theory with Lippmann's theory and derived the relationship between contact angle (CA) modulation and the applied electric potential [27]. By further coating a hydrophobic layer on top of the dielectric layer, the aqueous solution can achieve a wide range of reversible CA modulation, approximately from 110° to 65° [8, 29]. This wide, reversible modulation of CA enabled by EWOD presents various opportunities for applications [30, 31, 32]. Consequently, extensive research on EWOD has been conducted in fields such as digital microfluidics (DMF) [33], liquid lenses [34], display devices [19, 35], and others [36, 37, 38], leading to significant progress and promising prospects for commercialization [39].

The microfluidic manipulation of liquids through electrowetting was initially demonstrated using mercury droplets in water [40] and later extended to systems involving water in air [41] and water in oil [42]. The subsequent realization of two‐dimensional droplet path control in the early 21st century [43] marked the emergence of DMF. On a DMF platform, patterned electrodes are sequentially activated to asymmetrically modulate the CA of a microdroplet, generating a Laplace pressure difference that drives its motion [26], as illustrated in Figure 1b. Based on this principle, basic droplet operations such as transport, splitting [44], dispensing [45], merging, and parallel actuation of multiple droplets have been demonstrated [46]. Compared with flow‐through microfluidics, DMF offers advantages including the ability to handle smaller sample volumes, improved tolerance to a variety of solvents [8], facile automation, and feasibility for multi‐step reactions. These merits have led to widespread exploration and initial commercialization of DMF technologies in applications such as single‐cell analysis [47], immunoassays [48], DNA amplification [49], enzymatic assays, and radiochemical synthesis [50], among others [51].

Despite these remarkable achievements, Conventional electrowetting (CEW) based DMF actuates droplets through stepwise, electrode‐by‐electrode switching driven by local contact angle modulation. While this paradigm enables excellent addressability and supports a rich toolkit of droplet operations (e.g., dispensing, transport, merging, splitting, and mixing) [33, 52], scaling CEW‐DMF to long‐range routing and high parallel throughput often incurs increased electrode count, interconnect density, and control/scheduling complexity at the system level. In many lab‐on‐a‐chip workflows, where droplet transport serves as the connective operation linking unit functions such as metering, mixing, incubation, washing, and detection, these architectural overheads can become practical bottlenecks. This motivates the question of whether droplets can be transported continuously by breaking the symmetry of the electrowetting field, rather than relying exclusively on sequential switching of spatially discrete electrodes. Recently, we reported orbital electrowetting (OEW), which employs an asymmetric electrowetting force to drive continuous droplet motion along electrode gaps. OEW enables continuous transport along a predefined orbit while simplifying addressing and circuit management, thereby providing a new strategy for transport‐centric tasks in DMF systems [53] (Figure 1c). On superhydrophobic surfaces, OEW achieves nearly five times faster transport speed, significantly improving efficiency. On slippery surfaces, OEW supports fundamental DMF operations such as droplet transport, mixing, and splitting, with good flexibility and controllability [54]. Table 1 compares CEW and OEW in terms of their performance for the DMF application. These insights highlight improvements in OEW and offer guidance for future research to address practical challenges through hybrid integration with CEW.

**TABLE 1:** Comparison of CEW and OEW for DMF application.

Aspect	CEW	OEW
Key innovation	General‐purpose DMF with fine addressability via pixelated electrodes	Fast long‐range transport over multiple droplet diameters using only a single electrode pair along a predefined orbit
Electrode addressing & wiring.	Multiple individually addressed electrodes; Wiring/interconnect and scheduling complexity can grow with routing length and parallelism	Minimal independently addressed electrodes (single‐pair drive), simplifying wiring and circuit management for transport pathways
Motion/driving paradigm	Stepwise translation through sequential switching of spatially discrete electrodes	Continuous transport driven by an asymmetric electrowetting field along orbital pathways
Electrode geometry & enabling conditions	Performance depends on electrode pitch, actuation sequence, and liquid‐solid interactions.	Specific orbit geometry (small tapering angle) combined with very low contact angle hysteresis enables robust continuous motion.
Transport performance	Effective and versatile; speed depends on switching frequency and hysteresis; long‐range routing may incur control overhead.	Higher transport speed demonstrated (e.g., ∼5 times on superhydrophobic substrates) with simplified control on dedicated orbits
Workflow integration	Broad DMF toolbox including on‐demand dispensing, transport, splitting, mixing, merging, etc.	Transport along predefined orbits, mixing and splitting demonstrated, while dispensing and broader workflow functions may be realized via hybrid integration with CEW workstations.
References	[33, 52]	[53, 54, 55]

The publication of research related to droplet transport has increased continuously over recent decades. A search on lens.org returns the number of publications displayed in Figure 1d, where the annual output in this area has grown substantially and is now approaching an exponential trend, highlighting the strong and rapidly increasing global interest in droplet transport. As shown in Figure 1e, publications on electrowetting have also exhibited a steady increase over the past decades, indicating sustained attention to this technique and growing interest from the research community. Despite these advances, OEW is still in its early stage of development, and several fundamental challenges remain to be addressed. Against this background, this perspective focuses on recent advances in OEW and its emerging applications. We summarize the underlying physical mechanisms, material choices, structural designs, and representative application scenarios of OEW. We then discuss the key limitations of current technologies and outline potential strategies to overcome these challenges. Finally, we provide an outlook on the integration of OEW into broader applications, including defogging in photovoltaic modules and enhanced freshwater harvesting when combined with radiative‐cooling technologies.

## 2 Mechanisms and Structures of OEW

In the CEW scheme, voltage is locally applied beneath a droplet through an electrode array. The electric field reduces the solid–liquid interfacial tension, resulting in a local decrease of CA according to the Young‐Lippmann relation,

(1) $$\cos\theta_{V}=\cos\theta_{0}+\frac{\varepsilon_{0}\varepsilon_{r}}{2\gamma_{\mathrm{LV}}d}V^{2}$$

where θ_V_ is the CA of the liquid, *V* is the electric potential between the liquid and the dielectric, ε_0_ is the vacuum dielectric constant, ε_r_ is the relative permittivity of the dielectric layer, *d* is the thickness of the dielectric layer, and γ_LV_ is the surface tension between liquid and gas. The droplet motion is achieved by sequentially switching voltages between neighboring electrodes, generating an apparent displacement of the contact line. Although CEW provides high precision and programmability, its reliance on sequential electrode actuation inherently confines the droplet to discrete positions, requiring complex circuitry. Moreover, the symmetric field distribution between activated electrodes cancels the time‐averaged lateral forces under alternating current (AC) excitation, preventing continuous motion. OEW was initially identified in our studies on EWOD droplet manipulation conducted on dual‐scale superhydrophobic surfaces [53]. EWOD droplet manipulation on superhydrophobic surfaces has long been constrained by the pinning effect. When voltage is applied, the CA of the droplet decreases, causing the liquid to become trapped within the microstructures of the superhydrophobic surface. As a result, the droplet cannot fully return to its initial state after the voltage is removed [56]. Building upon previous research [57], we designed a dual‐scale superhydrophobic surface featuring micron‐scale cavities and nanoscale structures. This surface achieved a droplet CA of ∼155° and a roll‐off angle of only ∼1.4°, demonstrating an ultra‐slippery property. Additionally, it enabled nearly complete reversibility in AC EWOD operation. A fully reversible Wenzel–to–Cassie–Baxter transition of the droplet under an AC voltage provides a critical foundation for achieving OEW‐driven droplet transport on superhydrophobic surfaces.

Additionally, the frequency of the applied AC voltage is a crucial factor governing OEW behavior. According to the classical description of AC electrowetting, the droplet response can be divided into several frequency regimes defined by the mechanical resonance frequency. ω_*m*_, and the electrical transition frequency ω_*c*_. At very low frequencies (ω < ω_*m*_), the droplet responds quasi‐statically, and its apparent CA and shape can largely follow the applied voltage. As the excitation frequency approaches ω_*m*_, hydrodynamic eigenmodes of the droplet may be resonantly excited, leading to strong oscillation, pronounced deformation, and complex internal flow [56, 58, 59, 60]. Both Tan et al. [53] and Lv et al. [61] consistently demonstrated that the applied AC voltage frequency governs the shift from conventional cross‐electrode electrowetting to OEW. In the intermediate regime above the mechanical‐response range but still below the electrical transition frequency (ω_*m*_ < ω < ω_*c*_), inertia prevents the droplet from following the AC excitation cycle by cycle, while the droplet still behaves electrically as a conductor. As a result, the electrowetting response is governed primarily by the time‐averaged Maxwell stress. In our system, this time‐averaged actuation, together with the spatial asymmetry introduced by the tapered electrode gap, drives the droplet longitudinally along the orbit, corresponding to the OEW mode. By contrast, low‐frequency actuation, especially near the hydrodynamic transition region, tends to induce strong oscillation and unstable motion, which may promote cross‐electrode translation rather than stable orbital transport, corresponding to the CEW regime. Within the intermediate frequency window of (∼70 Hz–5 kHz), stable, reversible, and high‐speed droplet transport is achieved on the superhydrophobic surface due to its ultralow friction. When the frequency approaches the electrical transition frequency ω_*c*_, the electric field begins to penetrate the droplet phase, reducing the interfacial Maxwell stress and gradually weakening the electrowetting effect, eventually suppressing OEW motion. Therefore, the applied AC frequency not only governs the transition between CEW and OEW regimes but also defines the operational limits for efficient droplet transport in OEW systems. For droplets on slippery surfaces or immersed in oil environments, the viscous dissipation near the contact line is significantly enhanced, which makes the droplets more resistant to detachment from their orbital positions. This increased damping alters the dynamic response of the system, potentially shifting the frequency window associated with the CEW‐to‐OEW transition. Consequently, the critical frequency delineating the boundary between CEW and OEW modes under such conditions remains an open question, requiring further experimental and theoretical investigation to elucidate how viscous dissipation, hydrodynamic damping, and surrounding medium properties influence the frequency‐dependent electrowetting dynamics.

**FIGURE 2:**
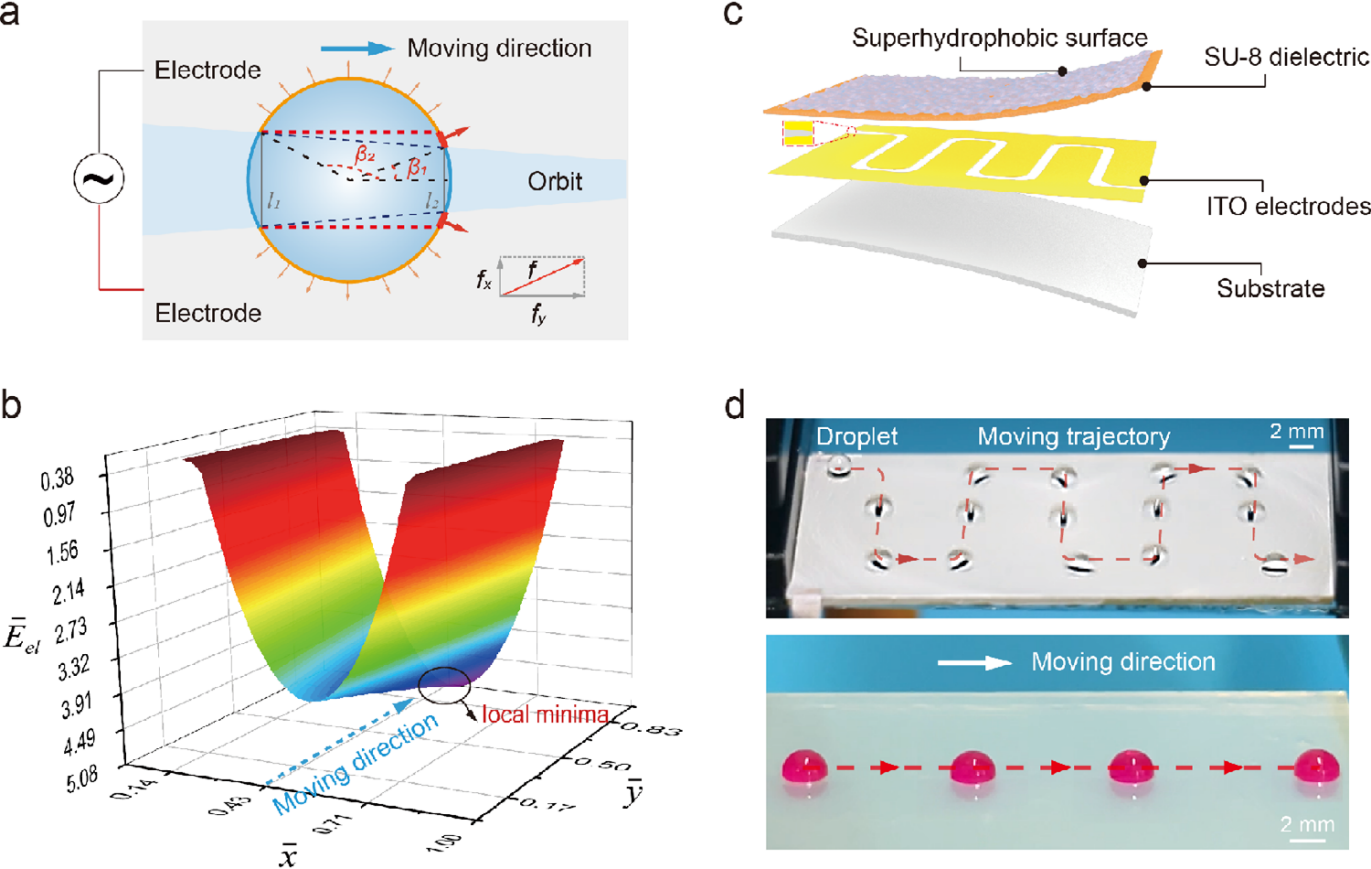
OEW mechanism and structural design. (a), Schematic representation of the asymmetric orbital structure and the forces exerted on the droplet. Copyright 2024 Wiley‐VCH. Reproduced with permission [53]. (b), Numerical simulation illustrating the electrostatic energy distribution of a droplet at various positions. Copyright 2025 Wiley‐VCH. Reproduced with permission [54]. (c), Schematic depiction of the OEW structure on a dual‐scale superhydrophobic surface. Copyright 2024 Wiley‐VCH. Reproduced with permission [53]. (d), Sequential images demonstrate OEW‐driven droplet manipulation on both superhydrophobic and slippery surfaces. Copyright 2024 Wiley‐VCH. Reproduced with permission [53]. Copyright 2025 Wiley‐VCH. Reproduced with permission [54].

With the increase in the applied AC voltage frequency, the droplet dynamics gradually approach a quasi‐steady state, as transient oscillations and interfacial perturbations are significantly reduced. Under this condition, when the minor variations at the droplet interface are neglected, a simplified dynamic model of OEW can be established to predict the droplet motion velocity. The model treats the droplet as a quasi‐static body driven by a time‐averaged, asymmetric electrowetting force along the orbit, providing a quantitative relationship between the applied voltage parameters, electrode geometry, and the droplet's velocity (as shown in Figure 2a). We established the droplet dynamic equations on both dual‐scale superhydrophobic and slippery surfaces, which demonstrate good agreement with experimental results [53, 54]. The model accurately predicts the acceleration and deceleration behavior of droplets moving along tapered orbits. However, the experimental observation that droplets can achieve continuous steering along arc‐shaped orbits suggests that further detailed experimental investigations and theoretical analyses are necessary to develop a more comprehensive model and description of OEW droplet motion. Additionally, the finite element method (FEM) can be used to calculate the electrostatic energy $E_{el}$ profile over an OEW unit. By continuously varying the droplet position in COMSOL Multiphysics and performing spatial integration, the distribution of the system's electrostatic energy at different droplet locations, denoted as $E_{el}\left(x,y\right)$, following approaches described in earlier studies, including Mannetje et al. [62], and in later related work [53, 54, 63]. As shown in Figure 2b, the corresponding mapping illustrates the nondimensionalized electrostatic energy landscape, expressed as ${\bar{E}}_{el}=\left|E_{el}\right|/\left(4\pi r^{2}\gamma_{LV}\right)$, where *r* is the radius of the droplet. The system exhibits the lowest electrostatic energy when the droplet is located in the narrower region of the orbit, indicating that the direction of droplet motion is governed by the orbital geometry. In addition, the minimum energy occurring at the center of the orbit explains why the droplet tends to travel along the orbital path rather than deviating from it. It should be noted that, in this analysis, the droplet is approximated as a non‐deformable and non‐flowing body, which corresponds well to the condition under a high‐frequency AC voltage. Under low‐frequency excitation, however, strong droplet oscillations significantly complicate the modeling process, and more in‐depth and comprehensive studies are required to establish a unified description that captures the full frequency‐dependent behavior of OEW systems. The OEW device typically consists of a substrate, indium tin oxide (ITO) electrodes, a dielectric layer, and a hydrophobic surface, arranged sequentially from bottom to top [53]. The ITO electrodes are widely adopted in OEW studies owing to their high optical transmittance and ease of microfabrication. The SU‐8 dielectric layer is chosen for its favorable dielectric properties and simple processability, such as spin coating. Since OEW operation requires an extremely low‐friction interface, various surface configurations have been employed, including dual‐scale superhydrophobic surfaces, slippery liquid‐infused porous surfaces (SLIPS), and Teflon surfaces immersed in silicone oil environments [53, 54, 55, 64]. As illustrated in Figure 2c, the schematic shows the OEW device structure based on a dual‐scale superhydrophobic surface. The upper image in Figure 2d demonstrates OEW droplet transport on this dual‐scale surface, where continuous and rapid long‐distance motion, achieving ten successive directional turns, is realized using only a single pair of electrodes, with a velocity approximately five times higher than that of CEW‐based droplet transport. The lower image in Figure 2d presents OEW droplet transport on a SLIPS surface, exhibiting enhanced control precision, broader liquid compatibility, and more versatile functionality compared to superhydrophobic surfaces [54].

## 3 OEW Droplet Manipulation

### 3.1 OEW Droplet Manipulation on a Dual‐Scale Superhydrophobic Surface

**FIGURE 3:**
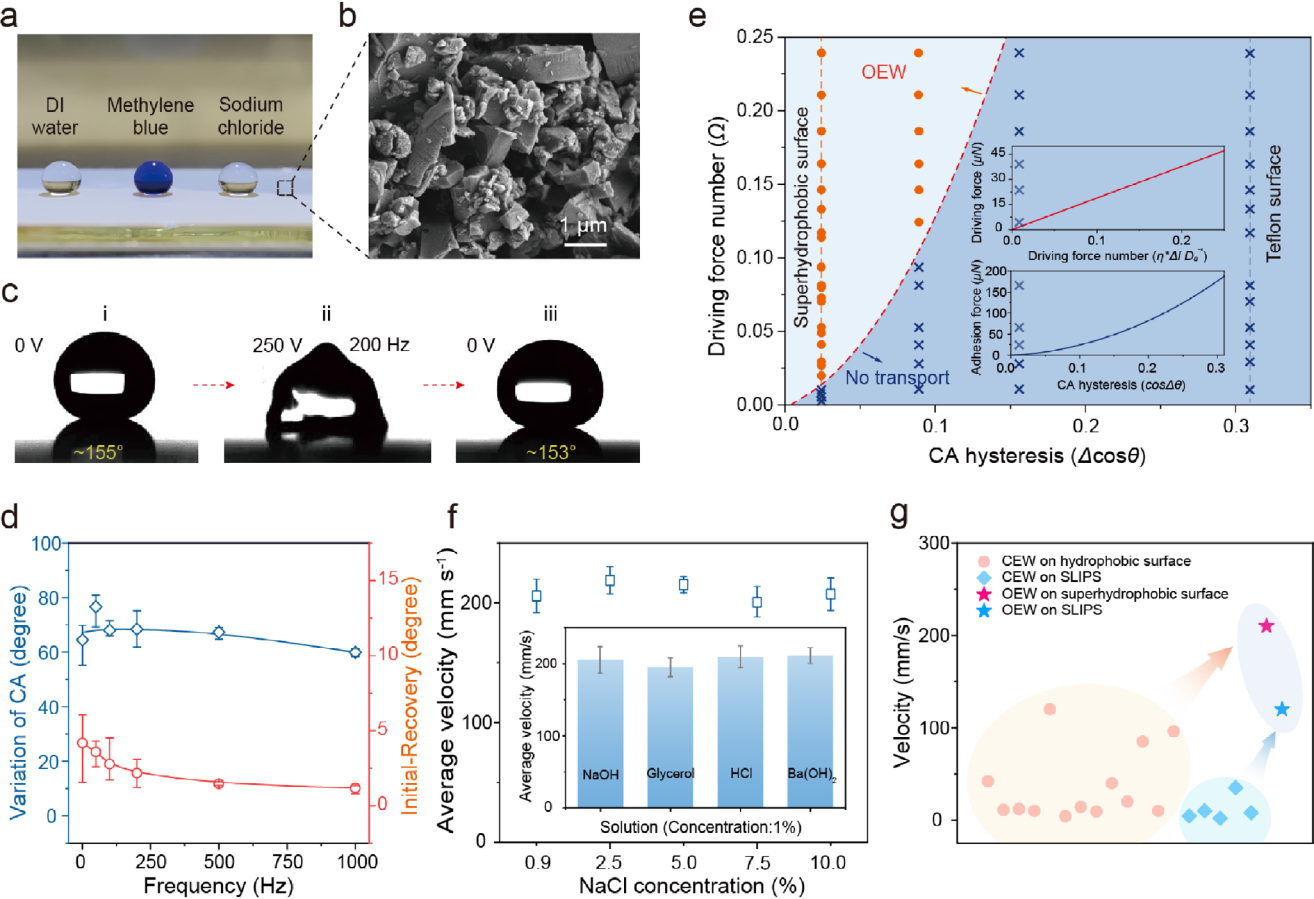
Dynamics of OEW droplet manipulation and characterization on a superhydrophobic surface. (a), Image of droplets of deionized (DI) water, methylene blue, and sodium chloride on a dual‐scale superhydrophobic surface. (b), SEM image showing the dual‐scale structure of the superhydrophobic surface. Scale bar, 1 µm. (c), Sequential images showing the variation of droplet CA under AC voltage on a dual‐scale superhydrophobic surface. (d), CA variation and recovery characteristics of the droplet under a 250 V AC voltage at different frequencies, showing excellent reversibility of CA modulation (10 µL droplet). (e), Phase diagram of OEW droplet manipulation on surfaces with different CA hysteresis. The horizontal axis denotes CA hysteresis, and the vertical axis represents the driving force number. Regions shaded in deep blue (symbols and background) correspond to conditions where the adhesion force induced by CA hysteresis dominates over the OEW driving force, leading to unsuccessful droplet actuation. The light‐blue region with orange symbols indicates successful OEW manipulation. Each data point corresponds to one experimental test. The insets schematically illustrate how the balance between adhesion force and OEW driving forces evolves across regimes. (f), The generality of droplet transportation for various liquids, demonstrating wide versatility in transporting acids, bases, organic solutions, and salt solutions with no reduction in velocity. Copyright 2024 Wiley‐VCH. Reproduced with permission (Figures a–f) [53]. (g), Comparison of the velocity of OEW droplet transport and CEW on different surfaces.

As shown in Figure 3a, the images of deionized (DI) water, methylene blue, and sodium chloride droplets on the dual‐scale superhydrophobic surface clearly demonstrate its excellent superhydrophobicity. The scanning electron microscopy (SEM) image reveals that the surface features large micro‐scale cavities, combined with nanoscale features, which collectively create abundant air pockets to sustain the lotus‐leaf‐like superhydrophobic effect. Meanwhile, this dual‐scale architecture enables the droplet to exhibit reversible apparent variations in CA under AC EWOD conditions. As shown in Figure 3c, a 10 µL DI water droplet exhibits an initial CA of approximately 155°. When an AC voltage with a frequency of 200 Hz and an amplitude of 250 V is applied, the CA decreases to a minimum of approximately 87.5°. Because the droplet is continuously actuated by the AC electric field, this configuration is transient rather than a static equilibrium state. After the voltage is removed, the droplet recovers to approximately 153°, indicating a highly reversible electrowetting response on the dual‐scale superhydrophobic surface. The reverse electrowetting behavior can occur over a wide frequency range, from 1 Hz to 1 kHz, without requiring specific parameter tuning. This observation demonstrates the universality of reversible AC electrowetting on the dual‐scale superhydrophobic surface, highlighting its robust and stable electromechanical response across a broad operational frequency spectrum (Figure 3d). Here, the reversibility refers to recovery after removal of the AC voltage, rather than reversible switching between two static wetting states [65].

The OEW droplet transport on a dual‐scale superhydrophobic surface exhibits extremely high velocity, where the droplet motion is governed by the balance between the driving force and the adhesion force. The driving force can be expressed as [53]

(2) $$F_{y}=\frac{1}{8d}\varepsilon_{0}\varepsilon_{r}\beta^{*}V^{2}\left(l_{1}-l_{2}\right)$$

where β* is the effective contact area fraction, *l*_1_ and *l*_2_ are the widths of the wider and narrower parts of the tapered orbit, respectively (as shown in Figure 2a). The lateral adhesion force caused by CA hysteresis is given by *F*_c_ =  ωγ_LV_ *k*(cosθ_r_ − cosθ_a_), where ω is the width of the contact area of the droplet, *k* ≈ 1 is a geometrical factor that depends on the detailed shape of the droplet, θ_*r*_ and θ_a_ are receding and advancing CAs, respectively. Therefore, the dynamic equation governing the OEW droplet motion can be expressed as

(3) $$m\frac{dv}{dt}=\frac{1}{8d}\varepsilon_{0}\varepsilon_{r}\beta^{*}V^{2}\left(l_{1}-l_{2}\right)-\omega {\gamma_{L}}_{V}k\left(\cos\theta_{r}-\cos\theta_{a}\right)$$

where *m* is the mass of the droplet, *v* is the velocity of the droplet, and *t* is time. The driving force is determined by the orbital gradient Φ and the electrowetting number η*, that is Ω  =  Φη*, while the resistive force depends on the CA  hysteresis [53]. As illustrated in Figure 3e, the phase diagram confirms that the proposed model accurately predicts the behavior of OEW droplets across surfaces with varying levels of contact angle hysteresis. The inset in Figure 3e further compares the variations in driving force and adhesion force, clarifying the balance that governs droplet motion. These results indicate that OEW operation is readily achieved on superhydrophobic surfaces with extremely low CA hysteresis (<0.05) but is difficult to realize on conventional hydrophobic surfaces, where the hysteresis exceeds 0.25 [53]. The dual‐scale superhydrophobic surface used exhibits a particularly small hysteresis of approximately 0.02 (obtained via cosine interpolation), ensuring highly efficient droplet transport [53]. The most distinctive feature of OEW droplet manipulation on superhydrophobic surfaces is its exceptionally high velocity, reaching approximately 210 mm/s, which is nearly five times faster than that achieved in CEW. Compared with other electric‐field‐based droplet control techniques [66, 67], OEW enables high‐speed transport of droplets with high ionic concentrations (e.g., up to 10 wt% NaCl solution) and maintains rapid motion for a wide range of liquids, including acids, bases, and salts, as shown in Figure 3f. In addition, the droplets exhibit a certain antigravity transport capability, achieving a maximum climbing angle of 55° and demonstrating stable operation over more than 1000 continuous cycles [53]. Furthermore, as shown in Figure 3g, OEW substantially enhances droplet transport velocity on different types of surfaces, highlighting the high‐efficiency droplet transport capability of this technique [53, 54, 68, 69, 70, 71, 72, 73, 74, 75, 76, 77, 78, 79, 80, 81, 82, 83, 84].

Benefiting from the low interfacial friction of the dual‐scale superhydrophobic surface, the OEW phenomenon was first discovered on this type of surface. As a novel EWOD‐based droplet manipulation technique, OEW on superhydrophobic interfaces has achieved significant breakthroughs in transport speed, complex trajectory control, and simplified electrode and operation design. However, several challenges remain for practical applications. For instance, precise droplet control in digital microfluidics, such as accurately maintaining droplets at designated locations for chemical or biological reactions, as well as the manipulation of complex biological or multi‐component samples, still requires further technological advancement and system optimization.

### 3.2 OEW Droplet Manipulation on SLIPS

**FIGURE 4:**
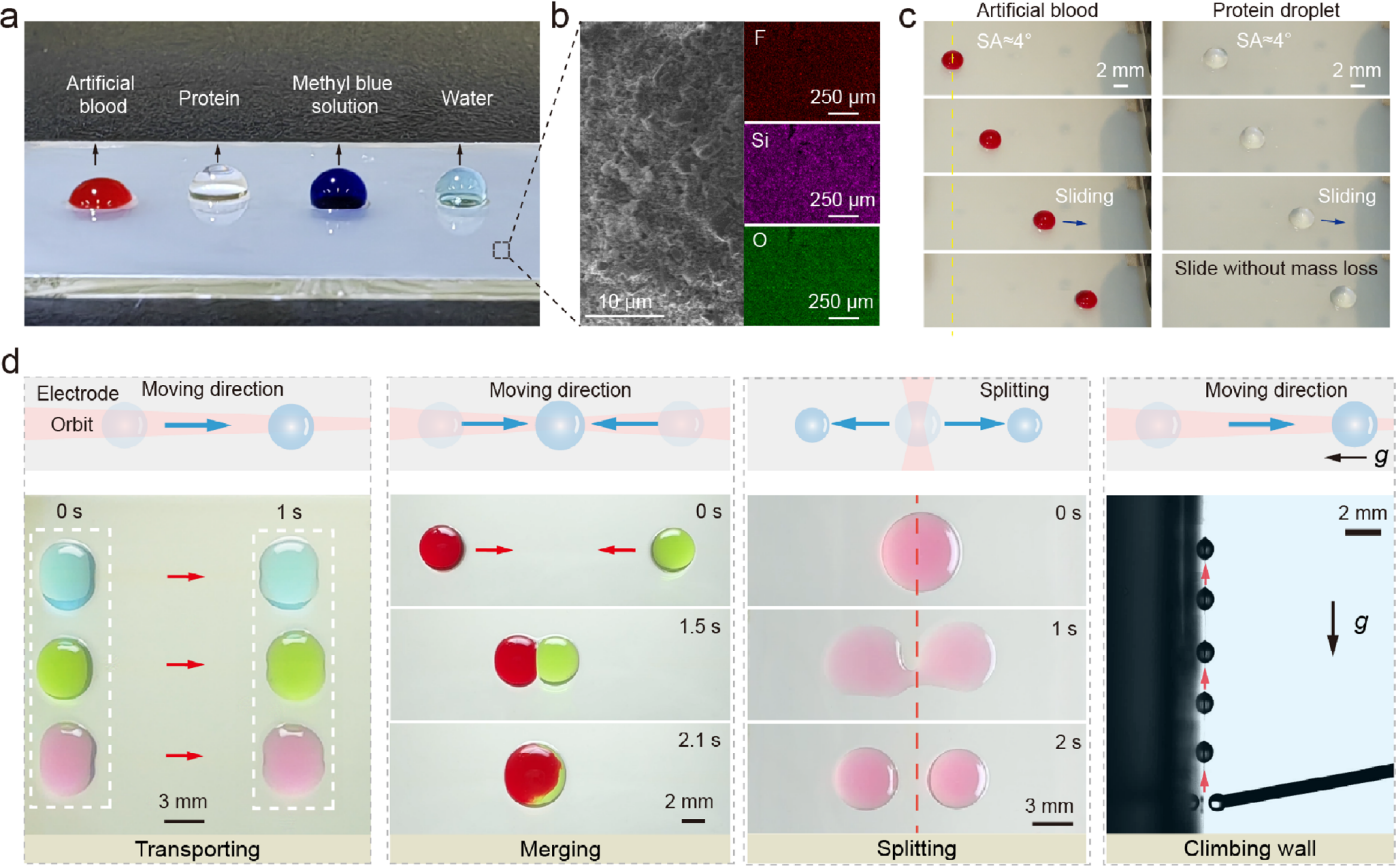
Dynamics of OEW droplet manipulation and characterization on SLIPS. (a), Image of droplets of artificial blood, protein, methylene blue, and DI water on a slippery surface. (b), SEM image showing the surface topography and energy‐dispersive x‐ray (EDX) spectroscopy mapping of the Krytox GPL 100 lubricated porous surface. (c), Sliding behavior of artificial blood and bovine serum albumin (BSA) droplets on a SLIPS, showing non‐adhesive characteristics and minimal retention force. Scale bar: 2 mm. (d), Multifunctional droplet manipulation enabled by OEW on a SLIPS, demonstrating transport, merging, splitting, and wall‐climbing capabilities. Copyright 2025 Wiley‐VCH. Reproduced with permission (Figures a–d) [54].

Inspired by *Nepenthes* pitcher plants, Wong et al. proposed the slippery liquid‐infused porous surface (SLIPS) concept [85]. Shortly thereafter, Hao et al. demonstrated reversible modulation of droplet CA under EWOD actuation on SLIPS [86], effectively eliminating the contact pinning encountered in CEW. Subsequently, Geng et al. demonstrated that EWOD on SLIPS enables residue‐free transport of various biological liquids, including protein solutions, sheep blood, and DNA solutions [87]. Therefore, extending the OEW technique to SLIPS is promising for residue‐free droplet manipulation, especially for biological samples. Meanwhile, the stability of the lubricant layer under electrowetting actuation deserves attention, since Maxwell‐stress‐induced thinning has been reported in related systems and may also influence OEW [88]. As shown in Figure 4a, the SLIPS was prepared by infiltrating Krytox GPL 100 oil into a porous membrane [54]. A DI water droplet placed on this surface exhibits a CA of approximately 115° and a roll‐off angle of about 2°, indicating ultra‐low adhesion. The SEM image of the SLIPS, together with the energy‐dispersive X‐ray (EDX) spectroscopy mappings, confirms that the oil uniformly and completely infiltrates the porous structure, as shown in Figure 4b. Figure 4c demonstrates the ultra‐low adhesion of artificial blood and bovine serum albumin (BSA) on the SLIPS surface, where complete sliding without any visible residue occurs at a tilt angle as small as 4°. Using this SLIPS surface, our OEW platform demonstrates residue‐free transport of biofluids such as BSA solutions and artificial blood, reliable manipulation of low surface tension liquids including ethanol and surfactant solutions (down to 22.39 mN/m), robust actuation of high–ionic strength media such as acidic, basic, and saline solutions (pH from 0.56 to 13.40 and ion concentrations up to 5.0 m), and the handling of droplets over a wide volume range from 0.5 µL to 1.0 mL, while maintaining transport speeds as high as 120 mm/s. For the same porous membrane, the typical actuation voltage decreases from about 250 V before oil infusion to about 150 V after forming SLIPS, indicating a lower actuation requirement than in the no‐oil case. A plausible interpretation is that the combination of the lower actuation voltage and the fast droplet motion helps preserve effective lubrication during transport, because droplet motion may occur on a shorter timescale than significant lubricant drainage. This mechanistic aspect deserves further systematic investigation. Compared with OEW transport on superhydrophobic surfaces, OEW on SLIPS introduces an additional friction force at the droplet foot, *F*_e_ ≈ 2πγ_ow_ *R*(η_o_ *v*/γ_ow_)^2/3^, where γ_ow_ is the surface tension of oil and water, *R* is the droplet radius, η_o_ is the oil viscosity and *v* is the droplet velocity. Because *F*_e_ increases with *v*, the overall resistance is small at the onset of motion but grows as the droplet speeds up. Such velocity‐dependent friction facilitates rapid droplet initiation, while providing larger resistance when the droplet approaches a target position, thereby enabling more accurate stopping and positioning [54].

Furthermore, as illustrated in Figure 4d, the appropriate design of the orbital tracks enables a variety of droplet operations, including multichannel droplet transport, droplet merging, droplet splitting, and even vertical uphill climbing. These capabilities lay the foundation for constructing OEW‐based digital microfluidic platforms. The droplet splitting observed here is consistent with previous reports of droplet splitting while crossing a gap between electrodes under AC electrowetting [89], which is primarily governed by electrostatic energy minimization. In our design, the slight taper mainly serves to localize the droplet position along the gap. Further study would be useful to quantify the splitting dynamics under the present OEW geometry. Moreover, the present OEW droplet transport scheme is essentially unidirectional, whereas practical microfluidic workflows often demand bidirectional droplet manipulation; this issue will be discussed in detail in Section 4.

### 3.3 Extensions and Recent Advances in OEW

**FIGURE 5:**
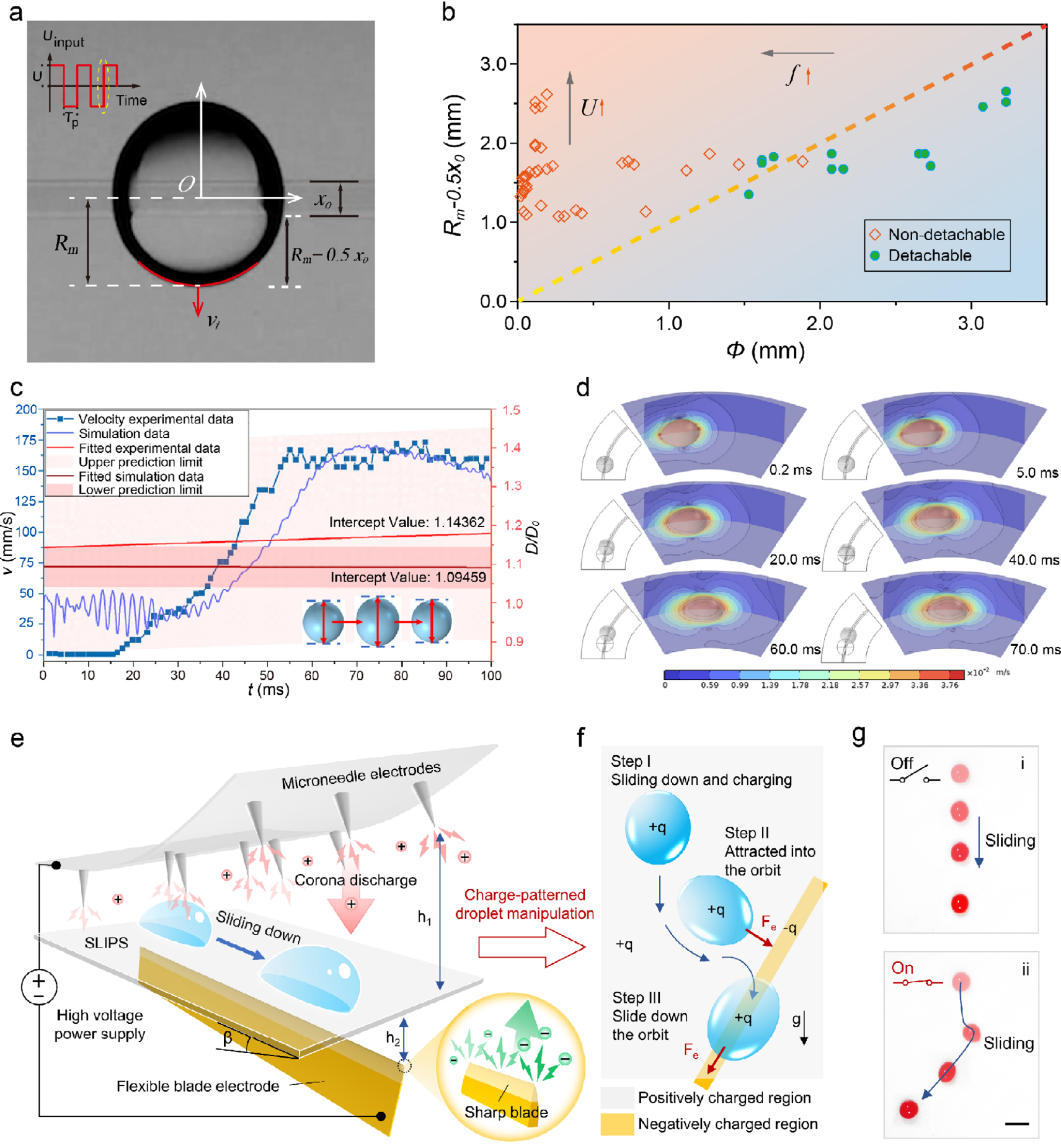
Critical transition and reconfigurable OEW on different slippery platforms. (a), High‐speed snapshot of a droplet on the orbit, indicating the orbit width *x*_0_, the maximum spreading radius *R_m_*, the contact‐line velocity *v_i_*, and the initial droplet position at the orbit center *O*. Copyright 2025 American Institute of Physics. Reproduced with permission [55]. (b), Phase diagram of *R_m_* − 0.5*x*_0_ versus the measured contact‐line displacement. Orange hollow diamonds denote droplets that remain confined on the orbit, while green solid circles denote droplets that detach. Copyright 2025 American Institute of Physics. Reproduced with permission [55]. (c), Comparison between measured and simulated OEW droplet velocity and spreading on a superhydrophobic surface, showing good agreement between experiment and phase‐field simulation. Copyright 2025 American Chemical Society. Reproduced with permission [55]. (d), Motion pattern and velocity field of a droplet traveling along a circular‐arc OEW orbit, illustrating the characteristic spread–shrink–advance behavior. Copyright 2025 American Chemical Society. Reproduced with permission [55]. (e), Schematic of ROEW on inclined SLIPS, where corona discharge from microneedle and blade electrodes non‐contactly prints charge‐defined orbits on the lubricated surface. Reproduced from ref. [64]. Copyright 2024 by the authors. Published by MDPI. (f), Working principle of ROEW. Reproduced from ref. [64]. Copyright 2024 by the authors. Published by MDPI. (g), Demonstration of pathway reconfiguration on SLIPS, showing straight gravitational sliding in the absence of an active blade electrode and curved, orbit‐guided transport when the underlying electrode is energized. Reproduced from ref. [64]. Copyright 2024 by the authors. Published by MDPI.

Beyond OEW operation on dual‐scale superhydrophobic surfaces and SLIPS, OEW has recently been extended and refined through both experimental and numerical studies on different substrates and electrode configurations. Building on our earlier OEW demonstrations, we systematically investigated the transition behavior between CEW and OEW on smooth Teflon surfaces immersed in silicone oil [55]. In this work, a water droplet on a Teflon surface was actuated by a pair of coplanar electrodes under AC square‐wave voltages. Two distinct motion modes were identified: a deviation mode, where the droplet detaches from the coplanar zone and exhibits cross‐electrode motion characteristic of CEW, and an oscillation mode, where the droplet remains confined within and oscillates along the orbit, which is the basis for OEW. By quantifying the spreading radius, contact‐line displacement, and capillary waves with high‐speed imaging, we derived a critical condition for droplet detachment (Figure 5a). This criterion links voltage amplitude and frequency to orbital stability, showing that sufficiently high frequencies suppress large contact‐line excursions and favor stable OEW (Figure 5b). Guided by this understanding, we designed tapered coplanar electrodes and, for the first time, realized OEW‐based droplet transport on a smooth Teflon surface in an oil environment, thereby complementing OEW on superhydrophobic surfaces and SLIPS.

Complementary theoretical insights have been provided by Lv et al. [61], who conducted a phase‐field simulation of OEW droplets on superhydrophobic surfaces. Starting from the existing OEW driving‐force model and our experimental data on dual‐scale superhydrophobic surfaces, they introduced an eccentricity correction factor into the electrowetting force to account for droplets whose center of mass does not coincide with the center of the orbit. Using COMSOL Multiphysics, they simulated the motion of 10 µL droplets along straight, inclined, and circular‐arc orbits, explicitly including off‐center configurations. The simulations reproduced key experimental features, such as a modest increase of spreading radius (∼1.09 times the initial radius), side ripples and internal recirculation during the early spreading stage, and a worm‐like “spread‐shrink‐advance” traveling mode at steady OEW. On inclined orbits, Lv et al. identified a maximum climb angle of ∼9.8° that OEW droplets can overcome against gravity, and on circular‐arc orbits, they quantified the steady off‐center bias and corresponding centripetal force, in good agreement with experimental estimates (Figure 5c,d). Overall, this work refines the OEW force model under eccentric and curved‐track conditions, providing a powerful numerical framework for designing more complex OEW circuits.

In parallel, Wu et al. [64] proposed a reconfigurable orbital electrowetting (ROEW) strategy for controllable droplet transport on inclined SLIPS (Figure 5e). Instead of using patterned contact electrodes, ROEW combines non‐contact corona‐discharge charge printing with flexible, deformable electrodes placed beneath the SLIPS. Asymmetrically arranged microneedle and blade electrodes generate positive and negative charges that are deposited non‐contact onto the porous, oil‐infused substrate, forming negatively charged “charge‐orbit” patterns on an otherwise positively charged background (Figure 5f). Positively charged droplets sliding down the inclined SLIPS are electrostatically attracted into these negatively charged orbits and then guided along them, enabling orbital‐like, pathway‐defined transport at speeds of up to ∼75 mm/s (Figure 5g). Because the charges can be rapidly erased and rewritten, and the flexible copper‐foil electrodes can be reversibly deformed and repositioned, ROEW offers a high degree of reconfigurability. By simply reshaping or moving a single reusable electrode, the system can program straight, 90°‐bent, and S‐shaped pathways on demand. Furthermore, by constructing composite electrodes with integrated switches, Wu et al. [64] achieved dynamically reconfigurable operations, such as online droplet sorting (utilizing Y‐shaped fork switches) and temporal mixing (employing V‐shaped electrodes with movable pin switches), all without the need for fixed electrode arrays beneath the droplet. Katoh et al. [90] recently reported a gravity‐driven electrowetting scheme that is conceptually similar to the approach of Wu et al. [64], in which gravity provides the primary propulsion, while electrowetting‐defined orbital paths confine and guide droplet motion, thereby extending the OEW paradigm further.

Taken together, these recent advances illustrate the rapid diversification of OEW. Our work on Teflon in oil establishes the critical transition conditions between CEW and OEW and demonstrates OEW transport on smooth, oil‐immersed surfaces. Lv et al. provide a detailed numerical description of OEW dynamics, especially under eccentric and curved orbits. Wu et al. extend the OEW concept to reconfigurable, non‐contact charge‐defined orbits on SLIPS, enabling programmable pathways and dynamic decision‐making. These developments collectively broaden the design space of OEW, which will be discussed in Section 4, along with the challenges and opportunities that arise.

## 4 Future Outlook of OEW

**FIGURE 6:**
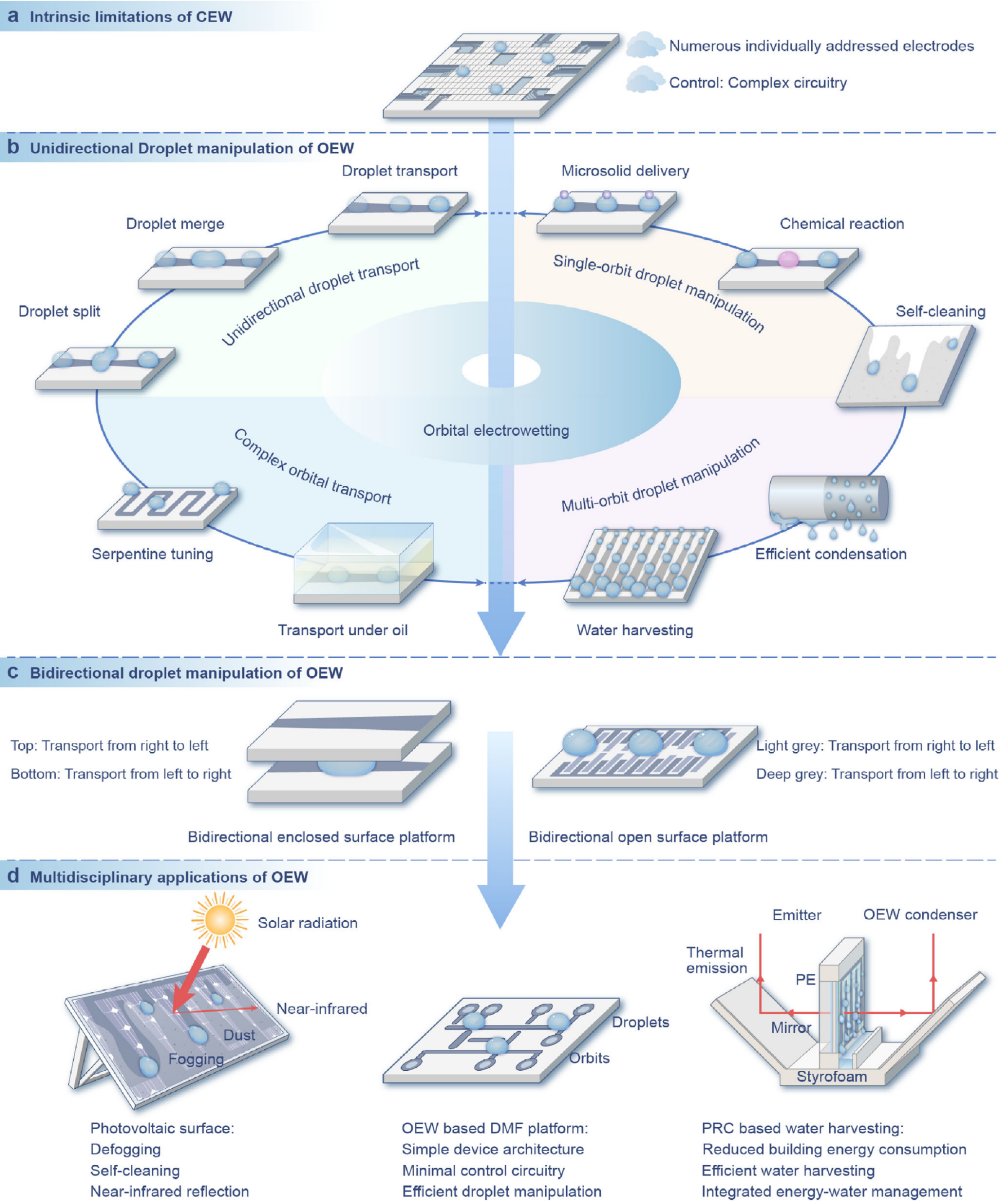
OEW platforms and their multidisciplinary applications. (a), Intrinsic limitations of CEW‐based DMF, which require numerous individually addressed electrodes and complex control circuitry. (b), Unidirectional OEW droplet manipulation on open surfaces, enabling continuous orbital transport and basic functions such as droplet transport, splitting, merging, microsolid delivery, chemical reactions, self‐cleaning, transport under oil, serpentine turning, and water harvesting. (c), Bidirectional OEW droplet manipulation on enclosed and open surface platforms. (d), Representative multidisciplinary applications of OEW, including defogging and self‐cleaning on photovoltaic surfaces, an OEW‐based DMF platform with simple architecture and minimal circuitry, and a PRC‐assisted OEW system for water harvesting with reduced building energy use and integrated energy‐water management.

As shown in Figure 6a, the emergence of OEW offers a promising route to overcome two intrinsic limitations of CEW, namely the requirement of numerous individually addressed electrodes (for example, CEW platforms have been reported with in the order of 10^2^ electrodes on a single chip [47, 51]) and complex control circuitry. As shown in Figure 6b, on a single orbit, an appropriate orbital design enables the unidirectional transport of droplets on open surfaces, as well as droplet merging, splitting, motion along serpentine paths with turns, and relatively precise stop–lock–restart control. These functions can be maintained even in encapsulated configurations, such as silicone oil, thereby overcoming the constraints of CEW in terms of low speed, large electrode count, and complicated driving schemes. Furthermore, extending from single to multiple orbits has revealed the potential of OEW for surface cleaning, micro‐chemical reactors, enhanced heat transfer, and freshwater collection (potentially complementing related electrowetting‐based water‐collection studies such as that of Hoek et al. [91] through continuous orbital droplet transport). On the mechanistic side, recent studies have established models that account for surface wettability, electric‐field distribution, orbital geometry, and viscous dissipation, deriving scaling relations between droplet velocity and parameters such as applied voltage and orbital gradient. Electrostatic energy simulations show that gradient electrodes create “energy valleys” along the orbit, toward which droplets spontaneously migrate and within which they remain confined. The experimentally measured evolution of droplet velocity closely agrees with theoretical predictions, providing a systematic physical foundation for the high‐speed and stable manipulation enabled by OEW.

However, existing demonstrations have thus far realized only unidirectional transport. For OEW to be successfully implemented in practical microfluidic platforms, more precise control over droplet position is required, and the capability for fully bidirectional motion is a key missing element. Building on prior work, we propose two solutions, as illustrated in Figure 6c. In the first, a sandwich‐type OEW chip is designed with oppositely graded orbits on the top and bottom plates, with the droplet confined between them (Figure 6c, left). By simply adding one additional switching element, bidirectional motion can be achieved. When a voltage is applied to the bottom chip, the droplet moves from left to right, whereas activation of the top chip drives motion in the opposite direction. In the second, analogous functionality can be realized on an open platform (Figure 6c, right). The electrode consists of two sets of OEW orbits with opposite gradient directions, interdigitated on the same plane. When the dark‐grey electrodes are energized, the droplet moves from left to right; activating the light‐grey electrodes reverses the direction of transport. A related open‐surface implementation could also employ two adjacent electrode gaps with opposite tapering directions, allowing the droplet to move along one gap and then laterally transfer to the other for reverse motion.

Once robust bidirectional and position‐resolved control is established, one can further exploit the multifunctionality of droplets to design OEW‐based microfluidic platforms tailored to specific operational requirements, potentially achieving substantial gains in both design cost and control efficiency. As shown in Figure 6d, beyond microfluidics, OEW also holds considerable promise in several interdisciplinary applications. One example is defogging and dust removal on photovoltaic (PV) surfaces (Figure 6d, left), where nighttime radiative cooling can cause heavy fogging on PV modules, reducing power output [92]. The strong multi‐droplet manipulation capability of OEW can significantly accelerate droplet removal and surface cleaning. In addition, ITO electrodes exhibit near‐infrared reflectivity (in a non‐photovoltaic active band), which can help passively cool PV modules during the daytime and further improve efficiency [93]. Similarly, combining OEW with passive radiative cooling (PRC) offers a pathway to enhance atmospheric water harvesting [54] (Figure 6d, right). Current collectors typically rely on static superhydrophobic or SLIPS surfaces, which struggle to simultaneously optimize rapid droplet nucleation and efficient droplet removal [10, 94]. OEW enables dynamic wettability control, potentially boosting both nucleation and collection efficiency. By integrating OEW with PRC, it may be possible to develop new strategies for building energy savings and water harvesting, thereby contributing to urban energy conservation and carbon reduction.

## 5 Conclusion

In summary, OEW converts asymmetric electrowetting forces and electrostatic energy gradients into continuous, high‐speed droplet motion confined along defined orbital pathways, while requiring only a minimal number of electrodes and simple driving schemes. Compared with conventional EWOD, OEW alleviates the long‐standing issues of densely pixelated electrodes, complex addressing circuitry, and step‐wise motion limited by contact‐angle hysteresis. In this perspective, we have outlined the underlying physical mechanisms of OEW, discussed material and structural design principles, and surveyed emerging applications ranging from microfluidics and thermal management to water harvesting.

Despite these advantages, OEW is still in its early stages, and several key challenges remain. Current demonstrations predominantly realize unidirectional transport on single or a few orbits, and truly bidirectional and position‐resolved droplet control in complex networks has yet to be established. The design of orbital geometries and field distributions is largely empirical, with limited support from systematic modeling and optimization. Issues such as multi‐droplet interactions, long‐term stability of lubricated or coated surfaces, charge accumulation, and large‐area manufacturability also need to be addressed before OEW can be translated into robust platforms.

Looking forward, we believe that the next phase of OEW research should focus on three intertwined directions. First, deeper physical understanding is needed through multiple‐physics models that couple electrowetting, wettability, viscous dissipation, and, where relevant, lubricant dynamics, enabling predictive design maps for droplet speed, stability, and capture criteria. Second, at the device level, realizing fully bidirectional, multi‐orbit routing with minimal electrodes and global driving is essential for OEW to evolve from proof‐of‐concept demonstrations into a programmable microfluidic platform. Third, targeted application‐oriented studies, such as defogging and self‐cleaning of photovoltaic modules, condensation heat transfer enhancement, and atmospheric water harvesting, are required to quantify the practical gains that OEW can offer relative to existing passive or CEW‐based technologies. With its unique combination of continuous high‐speed transport, low wiring complexity, and compatibility with open and encapsulated interfaces, OEW has the potential to reshape how droplets are actuated and routed on surfaces. We anticipate that, as the above challenges are addressed, OEW will complement and extend CEW, providing a versatile platform for next‐generation microfluidics, thermal management, and water‐energy systems.

## Author Contributions

Chi Yan Tso contributed to conceptualization, supervision, and writing – review and editing. Zuankai Wang contributed to the investigation and writing, review, and editing. Yikui Gao contributed to the investigation. Dongyue Jiang contributed to conceptualization, writing – original draft, and writing – review and editing. Jiayu Du contributed to writing – original draft. Dong Lv contributed to the investigation. Jie Tan contributed to conceptualization, investigation, writing – original draft, and writing – review and editing. Wenjie Liu contributed to writing – review, and editing.

## Conflicts of Interest

The authors declare no conflict of interest.

## Data Availability

The data that support the findings of this study are available from the corresponding author upon reasonable request.

## References

[1] H. Zhu , Q. Deng , J. Li , et al., “Sound‐Controlled Fluidic Processor,” Science Advances 11 (2025): adv6314, 10.1126/sciadv.adv6314. PMC12057661 40333956

[2] S. Liu , P. Sun , M. Wang , et al., “Acousto‐Dewetting Enables Droplet Microfluidics on Superhydrophilic Surfaces,” Nature Physics 21 (2025): 808–816, 10.1038/s41567-025-02844-6.

[3] Y. Fan , H. Wu , J. Wang , and J.‐A. Lv , “Field‐Programmable Topographic‐Morphing Array for General‐Purpose Lab‐on‐a‐Chip Systems,” Advanced Materials 37 (2025): 2410604, 10.1002/adma.202410604. 39555655

[4] G. Chen , Y. Wang , D. Jiang , et al., “A Programmable Compound Prism Powered by Triboelectric Nanogenerator for Highly Efficient Solar Beam Steering,” Nano Energy 80 (2021): 105524, 10.1016/j.nanoen.2020.105524.

[5] S. Yang , B. Ji , Y. Feng , et al., “How Surface Charges Affect Interdroplet Freezing,” Proceedings of the National Academy of Sciences 122 (2025): 2507849122, 10.1073/pnas.2507849122. PMC12207431 40531872

[6] W. Huang , L. Zhao , X. He , et al., “Low‐Temperature Leidenfrost‐Like Jumping of Sessile Droplets on Microstructured Surfaces,” Nature Physics 20 (2024): 1274–1281, 10.1038/s41567-024-02522-z.

[7] H. Wu , N. Mendel , S. Van Der Ham , L. Shui , G. Zhou , and F. Mugele , “Charge Trapping‐Based Electricity Generator (CTEG): An Ultrarobust and High Efficiency Nanogenerator for Energy Harvesting from Water Droplets,” Advanced Materials 32 (2020): 2001699, 10.1002/adma.202001699. 32627893

[8] J. Tan , S. Sun , D. Jiang , et al., “Advances in Triboelectric Nanogenerator Powered Electrowetting‐on‐Dielectric Devices: Mechanism, Structures, and Applications,” Materials Today 58 (2022): 201–220, 10.1016/j.mattod.2022.07.009.

[9] Z. Zhang , T. Li , Y. Yuan , et al., “A Self‐Sufficient System for Fog‐to‐Water Conversion and Nitrogen Fertilizer Production to Enhance Crop Growth,” Nature Communications 16 (2025): 4926, 10.1038/s41467-025-60340-0. PMC12117043 40425589

[10] I. Haechler , H. Park , G. Schnoering , et al., “Exploiting Radiative Cooling for Uninterrupted 24‐Hour Water Harvesting From the Atmosphere,” Science Advances 7 (2021): abf3978, 10.1126/sciadv.abf3978. PMC8221617 34162540

[11] J. Tan , H. Wang , M. Sun , et al., “Regulating Droplet Impact on a Solid Hydrophobic Surface Through Alternating Current Electrowetting‐on‐Dielectric,” Physics of Fluids 33 (2021): 042101, 10.1063/5.0044823.

[12] J. Tan , Z. Fan , S. Sun , M. Xu , and D. Jiang , “A Periodic Wetting Surface Driven by Triboelectric Nanogenerator for Efficient Postimpact Droplet Collection,” Advanced Materials Interfaces 10 (2023): 2202037, 10.1002/admi.202202037.

[13] J. Li , Y. Hou , Y. Liu , et al., “Directional Transport of High‐Temperature Janus Droplets Mediated by Structural Topography,” Nature Physics 12 (2016): 606–612, 10.1038/nphys3643.

[14] Q. Sun , D. Wang , Y. Li , et al., “Surface Charge Printing for Programmed Droplet Transport,” Nature Materials 18 (2019): 936–941, 10.1038/s41563-019-0440-2. 31332340

[15] H. Zhan , Z. Yuan , Y. Li , et al., “Versatile Bubble Maneuvering on Photopyroelectric Slippery Surfaces,” Nature Communications 14 (2023): 6158, 10.1038/s41467-023-41918-y. PMC10547833 37789018

[16] W. Lei , G. Hou , M. Liu , et al., “High‐Speed Transport of Liquid Droplets in Magnetic Tubular Microactuators,” Science Advances 4 (2018): aau8767, 10.1126/sciadv.aau8767. PMC6317984 30627667

[17] D. Jiang and S. Y. Park , “Light‐Driven 3D Droplet Manipulation on Flexible Optoelectrowetting Devices Fabricated by a Simple Spin‐Coating Method,” Lab on a Chip 16 (2016): 1831–1839, 10.1039/C6LC00293E. 27094708

[18] P. Sen and C. J. Kim , “A Liquid–Solid Direct Contact Low‐Loss RF Micro Switch,” Journal of Microelectromechanical Systems 18 (2009): 990–997, 10.1109/JMEMS.2009.2029170.

[19] R. A. Hayes and B. J. Feenstra , “Video‐Speed Electronic Paper Based on Electrowetting,” Nature 425 (2003): 383–385, 10.1038/nature01988. 14508484

[20] C. Sung Kwon , M. Hyejin , and C. J. Kim , “Creating, Transporting, Cutting, and Merging Liquid Droplets by Electrowetting‐Based Actuation for Digital Microfluidic Circuits,” Journal of Microelectromechanical Systems 12 (2003): 70–80, 10.1109/JMEMS.2002.807467.

[21] M. Paneru , C. Priest , R. Sedev , and J. Ralston , “Static and Dynamic Electrowetting of an Ionic Liquid in a Solid/Liquid/Liquid System,” Journal of the American Chemical Society 132 (2010): 8301–8308, 10.1021/ja9106397. 20507151

[22] F. Mugele , M. Duits , and D. Van Den Ende , “Electrowetting: A Versatile Tool for Drop Manipulation, Generation, and Characterization,” Advances in Colloid and Interface Science 161 (2010): 115–123, 10.1016/j.cis.2009.11.002. 20004880

[23] J. Lee , H. Moon , J. Fowler , T. Schoellhammer , and C. J. Kim , “Electrowetting and Electrowetting‐On‐Dielectric for Microscale Liquid Handling,” Sensors and Actuators A: Physical 95 (2002): 259–268, 10.1016/S0924-4247(01)00734-8.

[24] M. G. Pollack , A. D. Shenderov , and R. B. Fair , “Electrowetting‐Based Actuation of Droplets for Integrated Microfluidics,” Lab on a Chip 2 (2002): 96–101, 10.1039/b110474h. 15100841

[25] G. Lippmann , “Relations Entre Les Phénomènes Électriques et Capillaires,” Ann Chim Phys 11 (1875): 494–549.

[26] F. Mugele and J.‐C. Baret , “Electrowetting: from Basics to Applications,” Journal of Physics: Condensed Matter 17 (2005): R705–R774.

[27] B. Berge , “Electrocapillarité et Mouillage de Films Isolants Par L'eau,” Comptes Rendus de L'Academie des Sciences Paris, Serie, II 317 (1993): 157–163.

[28] H. Moon , S. K. Cho , R. L. Garrell , and C. J. Kim , “Low Voltage Electrowetting‐On‐Dielectric,” Journal of Applied Physics 92 (2002): 4080–4087, 10.1063/1.1504171.

[29] J. Tan , H. Zhang , M. Li , D. Jiang , and S. Sun , “Modeling and Observation of Fine Gas Compression in a Confined Narrow Tube by Electrowetting‐On‐Dielectric,” Physics of Fluids 34 (2022): 072113, 10.1063/5.0100611.

[30] P. Tian , M. Li , J. Tan , M. Sun , G. Chen , and D. Jiang , “Effects of Concave Curvature on the Formation and Propagation of Capillary Wave Induced by Electrowetting‐On‐Dielectric,” Physics of Fluids 34 (2022): 022104, 10.1063/5.0081362.

[31] P. Tian , L. Xia , J. Tan , M. Zhou , X. Yan , and D. Jiang , “Instability and Breakup of Rayleigh‐Plateau Jets with Capillary Wave Induced by Electrowetting‐On‐Dielectric,” Sensors and Actuators B: Chemical 418 (2024): 136197, 10.1016/j.snb.2024.136197.

[32] M. Zhou , J. Tan , Z. Han , X. Yan , and D. Jiang , “Nonconductive Droplet Oscillations Drive Flows Through Alternating Current Electrowetting,” Physics of Fluids 37 (2025): 072133, 10.1063/5.0273305.

[33] K. Choi , A. H. C. Ng , R. Fobel , and A. R. Wheeler , “Digital Microfluidics,” Annual Review of Analytical Chemistry 5 (2012): 413–440, 10.1146/annurev-anchem-062011-143028. 22524226

[34] S. Kuiper and B. H. W. Hendriks , “Variable‐Focus Liquid Lens for Miniature Cameras,” Applied Physics Letters 85 (2004): 1128–1130, 10.1063/1.1779954.

[35] F. Xu , W. Chen , B. Tang , and G. Zhou , “Inhibiting Oil Overflow in Electrowetting Display Through Selectively Hydrophilic Modification of the Pixel Wall Based on Polydopamine and Polydopamine/Polyvinylpyrrolidone via Regional Protection Strategy,” Advanced Materials Interfaces 10 (2023): 2300344, 10.1002/admi.202300344.

[36] D. Jiang , Z. Fan , H. Wang , et al., “Triboelectric Nanogenerator Powered Electrowetting‐on‐Dielectric Actuator for Concealed Aquatic Microbots,” ACS Nano 14 (2020): 15394–15402, 10.1021/acsnano.0c05901. 33179494

[37] C. Wang , X. Li , Y. Qiu , et al., “Electrowetting‐On‐Dielectric Powered by Triboelectric Nanogenerator,” Nano Energy 98 (2022): 107310, 10.1016/j.nanoen.2022.107310.

[38] H. Cui , Y. Song , D. Ren , L. Wang , and X. He , “Electrocapillary Boosting Electrode Wetting for High‐Energy Lithium‐Ion Batteries,” Joule 8 (2024): 29–44, 10.1016/j.joule.2023.11.012.

[39] J. Li and C. J. Kim , “Current Commercialization Status of Electrowetting‐On‐Dielectric (EWOD) Digital Microfluidics,” Lab on a Chip 20 (2020): 1705–1712, 10.1039/D0LC00144A. 32338272

[40] L. Junghoon and C. J. C. Kim , “Liquid Micromotor Driven by Continuous Electrowetting,” In Proceedings MEMS 98. IEEE. Eleventh Annual International Workshop on Micro Electro Mechanical Systems: An Investigation of Micro Structures, Sensors, Actuators, Machines and Systems Cat (IEEE, 1998).

[41] J.‐H. Lee , In Los Angeles ProQuest Dissertations & Theses (University of California, 2000).

[42] M. G. Pollack , R. B. Fair , and A. D. Shenderov , “Electrowetting‐Based Actuation of Liquid Droplets for Microfluidic Applications,” Applied Physics Letters 77 (2000): 1725–1726, 10.1063/1.1308534.

[43] G. Man and C. J. Kim , In 18th IEEE International Conference on Micro Electro Mechanical Systems (IEEE, 2005).

[44] N. Sagar , S. Bansal , and P. Sen , “Open‐Chip Droplet Splitting in Electrowetting,” Advanced Materials Interfaces 9 (2022): 2200240, 10.1002/admi.202200240.

[45] H. Ren , R. B. Fair , and M. G. Pollack , “Automated on‐Chip Droplet Dispensing with Volume Control by Electro‐Wetting Actuation and Capacitance Metering,” Sensors and Actuators B: Chemical 98 (2004): 319–327, 10.1016/j.snb.2003.09.030.

[46] W. C. Nelson and C.‐J. C. Kim , “Droplet Actuation by Electrowetting‐on‐Dielectric (EWOD): A Review,” Journal of Adhesion Science and Technology 26 (2012): 1747–1771, 10.1163/156856111X599562.

[47] Q. Ruan , W. Ruan , X. Lin , et al., “Digital‐WGS: Automated, Highly Efficient Whole‐Genome Sequencing of Single Cells by Digital Microfluidics,” Science Advances 6 (2020): abd6454, 10.1126/sciadv.abd6454. PMC7725457 33298451

[48] A. H. C. Ng , M. D. Chamberlain , H. Situ , V. Lee , and A. R. Wheeler , “Digital Microfluidic Immunocytochemistry in Single Cells,” Nature Communications 6 (2015): 7513, 10.1038/ncomms8513. PMC4491823 26104298

[49] Y.‐H. Chang , G.‐B. Lee , F.‐C. Huang , Y.‐Y. Chen , and J.‐L. Lin , “Integrated Polymerase Chain Reaction Chips Utilizing Digital Microfluidics,” Biomedical Microdevices 8 (2006): 215–225, 10.1007/s10544-006-8171-y. 16718406

[50] P. Y. Keng and R. M. van Dam , “Digital Microfluidics: A New Paradigm for Radiochemistry,” Molecular Imaging 14 (2015): 7290, 10.2310/7290.2015.00030. PMC4734895 26650206

[51] J. Zhai , Y. Liu , W. Ji , et al., “Drug Screening on Digital Microfluidics for Cancer Precision Medicine,” Nature Communications 15 (2024): 4363, 10.1038/s41467-024-48616-3. PMC11111680 38778087

[52] R. B. Fair , “Digital Microfluidics: is a True Lab‐On‐a‐Chip Possible?,” Microfluidics and Nanofluidics 3 (2007): 245–281, 10.1007/s10404-007-0161-8.

[53] J. Tan , Z. Fan , M. Zhou , et al., “Orbital Electrowetting‐on‐Dielectric for Droplet Manipulation on Superhydrophobic Surfaces,” Advanced Materials 36 (2024): 2314346, 10.1002/adma.202314346. 38582970

[54] J. Tan , H. Li , X. Yan , M. Zhou , S. Sun , and D. Jiang , “Orbital Electrowetting for Versatile Droplet Maneuvering on Slippery Surfaces,” Droplet 4 (2025): 70001.

[55] J. Tan , H. Li , M. Zhou , et al., “Critical Transition Conditions From Conventional to Orbital Electrowetting of a Water Droplet in Oil Environment,” Physics of Fluids 37 (2025): 062107, 10.1063/5.0264993.

[56] J. M. Oh , S. H. Ko , and K. H. Kang , “Shape Oscillation of a Drop in ac Electrowetting,” Langmuir 24 (2008): 8379–8386, 10.1021/la8007359. 18582134

[57] E. N. A. Latip , L. Coudron , M. B. McDonnell , et al., “Protein Droplet Actuation on Superhydrophobic Surfaces: a New Approach Toward Anti‐Biofouling Electrowetting Systems,” RSC Advances 7 (2017): 49633–49648, 10.1039/C7RA10920B.

[58] F. J. Hong , D. D. Jiang , and P. Cheng , “Frequency‐Dependent Resonance and Asymmetric Droplet Oscillation Under ac Electrowetting on Coplanar Electrodes,” Journal of Micromechanics and Microengineering 22 (2012): 085024, 10.1088/0960-1317/22/8/085024.

[59] F. Mugele , J. C. Baret , and D. Steinhauser , “Microfluidic Mixing Through Electrowetting‐Induced Droplet Oscillations,” Applied Physics Letters 88 (2006): 204106, 10.1063/1.2204831.

[60] F. Mugele , A. Staicu , R. Bakker , and D. van den Ende , “Capillary Stokes Drift: a New Driving Mechanism for Mixing in AC‐Electrowetting,” Lab on a Chip 11 (2011): 2011–2016, 10.1039/c0lc00702a. 21526233

[61] C. Lv , T. Zhou , Y. Liu , L. Zhang , H. Zhao , and B. Si , “Motion Characteristics of Orbital Electrowetting‐on‐Dielectric Droplets on Superhydrophobic Surfaces,” Langmuir 41 (2025): 8934–8950, 10.1021/acs.langmuir.5c00256. 40138340

[62] D. t Mannetje , S. Ghosh , R. Lagraauw , et al., “Trapping of Drops by Wetting Defects,” Nature Communications 5 (2014): 3559, 10.1038/ncomms4559. PMC3996538 24721935

[63] D. Baratian , R. Dey , H. Hoek , D. Van Den Ende , and F. Mugele , “Breath Figures Under Electrowetting: Electrically Controlled Evolution of Drop Condensation Patterns,” Physical Review Letters 120 (2018): 214502, 10.1103/PhysRevLett.120.214502. 29883164

[64] J. Wu , H. Li , Y. Zhou , et al., “Reconfigurable Orbital Electrowetting for Controllable Droplet Transport on Slippery Surfaces,” Micromachines 16 (2025): 618, 10.3390/mi16060618. 40572338 PMC12195050

[65] G. Manukyan , J. M. Oh , D. Van Den Ende , R. G. H. Lammertink , and F. Mugele , “Electrical Switching of Wetting States on Superhydrophobic Surfaces: A Route Towards Reversible Cassie‐to‐Wenzel Transitions,” Physical Review Letters 106 (2011): 014501, 10.1103/PhysRevLett.106.014501. 21231746

[66] Y. Jin , W. Xu , H. Zhang , et al., “Electrostatic Tweezer for Droplet Manipulation,” Proceedings of the National Academy of Sciences 119 (2022): 2105459119, 10.1073/pnas.2105459119. PMC8764671 34992136

[67] W. Xu , Y. Jin , W. Li , et al., “Triboelectric Wetting for Continuous Droplet Transpor,” Science Advances 8 (2022): ade2085. 10.1126/sciadv.ade2085 PMC9770939 36542697

[68] N. Kumari , V. Bahadur , and S. V. Garimella , “Electrical Actuation of Electrically Conducting and Insulating Droplets Using Ac and Dc Voltages,” Journal of Micromechanics and Microengineering 18 (2008): 105015, 10.1088/0960-1317/18/10/105015.

[69] J.‐H. Chang , D. Y. Choi , S. Han , and J. J. Pak , “Driving Characteristics of the Electrowetting‐On‐Dielectric Device Using Atomic‐Layer‐Deposited Aluminum Oxide as the Dielectric,” Microfluidics and Nanofluidics 8 (2010): 269–273, 10.1007/s10404-009-0511-9.

[70] N. Rajabi and A. Dolatabadi , “A Novel Electrode Shape for Electrowetting‐Based Microfluidics,” Colloids and Surfaces A: Physicochemical and Engineering Aspects 365 (2010): 230–236, 10.1016/j.colsurfa.2010.01.039.

[71] J.‐H. Chang and J. J. Pak , “Twin‐Plate Electrowetting for Efficient Digital Microfluidics,” Sensors and Actuators B: Chemical 160 (2011): 1581–1585, 10.1016/j.snb.2011.09.011.

[72] F. Lapierre , M. Jonsson‐Niedziolka , Y. Coffinier , R. Boukherroub , and V. Thomy , “Droplet Transport by Electrowetting: Lets Get Rough!,” Microfluidics and Nanofluidics 15 (2013): 327–336, 10.1007/s10404-013-1149-1.

[73] C. Dong , T. Chen , J. Gao , et al., “On the Droplet Velocity and Electrode Lifetime of Digital Microfluidics: Voltage Actuation Techniques and Comparison,” Microfluidics and Nanofluidics 18 (2015): 673–683, 10.1007/s10404-014-1467-y.

[74] Q. Ni , D. E. Capecci , and N. B. Crane , “Electrowetting Force and Velocity Dependence on Fluid Surface Energy,” Microfluidics and Nanofluidics 19 (2015): 181–189, 10.1007/s10404-015-1563-7.

[75] Y. Li , R. J. Baker , and D. Raad , “Improving the Performance of Electrowetting on Dielectric Microfluidics Using Piezoelectric Top Plate Control,” Sensors and Actuators B: Chemical 229 (2016): 63–74, 10.1016/j.snb.2016.01.108.

[76] M. M. Nahar , J. B. Nikapitiya , S. M. You , and H. Moon , “Droplet Velocity in an Electrowetting on Dielectric Digital Microfluidic Device,” Micromachines 7 (2016): 71, 10.3390/mi7040071. 30407443 PMC6189997

[77] I. Swyer , R. Fobel , and A. R. Wheeler , “Velocity Saturation in Digital Microfluidics,” Langmuir 35 (2019): 5342–5352, 10.1021/acs.langmuir.9b00220. 30958677

[78] J. Cao , Q. An , Z. Liu , et al., “Electrowetting on Liquid‐Infused Membrane for Flexible and Reliable Digital Droplet Manipulation and Application,” Sensors and Actuators B: Chemical 291 (2019): 470–477, 10.1016/j.snb.2019.04.102.

[79] K. Zhang , W. Wang , C. Li , A. Riaud , and J. Zhou , “2D Large‐Scale EWOD Devices with Honeycomb Electrodes for Multiplexed Multidirectional Driving of Micro‐Droplets,” AIP Advances 10 (2020): 055227, 10.1063/5.0008071.

[80] F. Qin , K. Zhang , B. Lin , et al., “Solution for Mass Production of High‐Throughput Digital Microfluidic Chip Based on a‐Si TFT with In‐Pixel Boost Circuit,” Micromachines 12 (2021): 1199, 10.3390/mi12101199. 34683251 PMC8541461

[81] E. Liu , C. Wang , H. Zheng , S. Song , A. Riaud , and J. Zhou , “Two‐Dimensional Manipulation of Droplets on a Single‐Sided Continuous Optoelectrowetting Digital Microfluidic Chip,” Sensors and Actuators B: Chemical 368 (2022): 132231, 10.1016/j.snb.2022.132231.

[82] T.‐J. Yang , Z.‐H. Lin , and Y.‐W. Lu , “Self‐Powered Digital Microfluidics Driven by Rotational Triboelectric Nanogenerator,” Nano Energy 110 (2023): 108376, 10.1016/j.nanoen.2023.108376.

[83] K. Yamamoto , S. Takagi , Y. Ichikawa , and M. Motosuke , “Lubrication Effects on Droplet Manipulation by Electrowetting‐On‐Dielectric (EWOD),” Journal of Applied Physics 132 (2022): 204701, 10.1063/5.0118241.

[84] S. Von Der Ecken , A. A. Sklavounos , and A. R. Wheeler , “Vertical Addressing of 1‐Plane Electrodes for Digital Microfluidics,” Advanced Materials Technologies 7 (2022): 2101251, 10.1002/admt.202101251.

[85] T.‐S. Wong , S. H. Kang , S. K. Y. Tang , et al., “Bioinspired Self‐Repairing Slippery Surfaces with Pressure‐Stable Omniphobicity,” Nature 477 (2011): 443–447, 10.1038/nature10447. 21938066

[86] C. Hao , Y. Liu , X. Chen , et al., “Electrowetting on Liquid‐Infused Film (EWOLF): Complete Reversibility and Controlled Droplet Oscillation Suppression for Fast Optical Imaging,” Scientific Reports 4 (2014): 6846, 10.1038/srep06846. 25355005 PMC4213809

[87] H. Geng and S. K. Cho , “Antifouling Digital Microfluidics Using Lubricant Infused Porous Film,” Lab on a Chip 19 (2019): 2275–2283, 10.1039/C9LC00289H. 31184676

[88] J. Gao , N. Mendel , R. Dey , D. Baratian , and F. Mugele , “Contact Angle Hysteresis and Oil Film Lubrication in Electrowetting With Two Immiscible Liquids,” Applied Physics Letters 112 (2018): 203703, 10.1063/1.5034510.

[89] R. de Ruiter , A. M. Pit , V. M. de Oliveira , M. H. G. Duits , D. Van Den Ende , and F. Mugele , “Electrostatic Potential Wells for On‐Demand Drop Manipulation in Microchannels,” Lab on a Chip 14 (2014): 883–891, 10.1039/c3lc51121a. 24394887

[90] K. Katoh , T. Wakimoto , J. Matsuura , and T. Ito , “Control of Sliding Droplet Movement Through Electrowetting on Dielectrics,” Physics of Fluids 37 (2025): 112116, 10.1063/5.0291779.

[91] H. Hoek , R. Dey , and F. Mugele , “Electrowetting‐Controlled Dropwise Condensation With Patterned Electrodes: Physical Principles, Modeling, and Application Perspectives,” Advanced Materials Interfaces 8 (2021): 2001317, 10.1002/admi.202001317.

[92] Y. Zhou , J. Wu , G. Gao , Y. Zeng , S. Liu , and H. Zheng , “Universal Droplet Propulsion by Dynamic Surface‐Charge Wetting,” Microsystems & Nanoengineering 10 (2024): 134, 10.1038/s41378-024-00745-x. 39327423 PMC11427456

[93] X. Jiao , S. Li , Z. Lv , H. Jiao , J. He , and J. Song , “Advances of Indium Tin Oxide in Catalysis and Cell,” Materials Today Communications 44 (2025): 112058, 10.1016/j.mtcomm.2025.112058.

[94] S. Ahmad , A. R. Siddiqui , K. Yang , et al., “Lubricated Surface in a Vertical Double‐Sided Architecture for Radiative Cooling and Atmospheric Water Harvesting,” Advanced Materials 36 (2024): 2404037, 10.1002/adma.202404037. 39239994

